# Lung cancer mutation testing: a clinical retesting study of agreement between a real-time PCR and a mass spectrometry test

**DOI:** 10.18632/oncotarget.21023

**Published:** 2017-09-16

**Authors:** Phillip Shepherd, Karen L. Sheath, Sandar Tin Tin, Prashannata Khwaounjoo, Phyu S. Aye, Angie Li, George R. Laking, Nicola J. Kingston, Christopher A. Lewis, J. Mark Elwood, Donald R. Love, Mark J. McKeage

**Affiliations:** ^1^ Auckland Uniservices, University of Auckland, Auckland 1142, New Zealand; ^2^ Diagnostic Genetics, LabPlus, Auckland City Hospital, Auckland 1148, New Zealand; ^3^ School of Population Health, University of Auckland, Auckland 1142, New Zealand; ^4^ Pharmacology and Clinical Pharmacology and Auckland Cancer Society Research Centre, University of Auckland, Auckland 1142, New Zealand; ^5^ Blood and Cancer Services, Auckland City Hospital, Auckland 1148, New Zealand; ^6^ Anatomical Pathology, LabPlus, Auckland City Hospital, Auckland 1148, New Zealand; ^7^ Respiratory Medicine, Auckland City Hospital, Auckland 1148, New Zealand

**Keywords:** agreement analysis, epidermal growth factor receptor, lung cancer, mutation testing, targeted therapy

## Abstract

To investigate the clinical validity and utility of tests for detecting Epidermal Growth Factor Receptor (*EGFR*) gene mutations in non-squamous non-small cell lung cancer patients, tumour DNA extracts from 532 patients previously tested by the cobas EGFR Mutation Test (RT-PCR test) were retested by the Sequenom/Agena Biosciences MassArray OncoFocus mass spectrometry test (MS test). Valid results from both tests were available from 470 patients (88%) for agreement analysis. Survival data were obtained for 513 patients (96%) and 77 patients (14%) were treated with EGFR tyrosine kinase inhibitors (TKIs). Agreement analysis revealed moderately high positive (79.8%), negative (96.9%) and overall percentage agreement (93.2%) for the detection of *EGFR* mutations. However, *EGFR* mutations were detected by one test and not by the other test in 32 patients (7%). Retesting of discordant samples revealed false-positive and false-negative results generated by both tests. Despite this, treatment and survival outcomes correlated with the results of the RT-PCR and MS tests. In conclusion, this study provides evidence of the clinical validity and utility of the RT-PCR and MS tests for detection of *EGFR* mutations that predict prognosis and benefit from EGFR-TKI treatment. However, their false-positive and false-negative test results may have important clinical consequences.

## INTRODUCTION

Epidermal Growth Factor Receptor (*EGFR*) gene mutation testing is a critical first step in the personalised treatment of patients with non-squamous non-small cell lung cancer (NSCLC). The testing is required for identifying patients with *EGFR* gene mutation-positive non-squamous NSCLC, who are candidates for first-line treatment with EGFR tyrosine kinase inhibitors (EGFR-TKIs). Significant numbers of non-squamous NSCLC patients can be expected to test positive for *EGFR* gene mutations, although the exact proportion varies widely between different ethnic groups and geographical regions. For example, in a population-based registry cohort of non-squamous NSCLC patients presenting in northern New Zealand, we had previously shown that *EGFR* gene mutations were detected in 109 of 500 tested patients (22%) [1]. Randomised trials had previously shown increased tumour response rates and progression-free survival with EGFR-TKI treatment in *EGFR* gene mutation-positive patients [2–8], and with platinum-doublet combination chemotherapy in *EGFR* gene mutation-negative patients [9, 10], who had previously untreated metastatic non-squamous NSCLC. In the same New Zealand population-based study mentioned above [1], we showed that the introduction of *EGFR* gene mutation testing was associated with improved quality of prescribing of EGFR-TKIs, and with improved health outcomes, including prolongation of overall survival and increased duration of benefit from EGFR-TKI treatment. Currently, no gold standard *EGFR* gene mutation testing methodology exists, and international clinical practice guidelines recommend use of any validated testing method with sufficient performance characteristics, but without recommending one or more individual methods to the exclusion of any others [11]. Surveys of real-world testing practices have revealed wide variation with the use of many different testing methodologies for *EGFR* gene mutation detection in the routine setting [12, 13].

The cobas EGFR Mutation Test (Roche Molecular Systems Inc., Branchburg, NJ, USA) (RT-PCR test) is an oncogene mutation detection protocol based on multiplexed allele-specific PCR and a pre-validated set of primers to amplify and detect 41 variant sequences in the tyrosine kinase domain (exons 18-21) of the *EGFR* gene [14]. This RT-PCR test achieved CE-IVD regulatory status in Europe in October 2011 and FDA-USIVA approval of a modified version of the test in April 2013 [15]. Clinical validation studies were undertaken by retrospective analyses of tumour specimens (often surgical) sourced from vendors or clinical trial collections, and the data compared to other testing methods [16–19].

The Sequenom/Agena Biosciences OncoFocus mass spectrometry test (Agena Bioscience, San Diego, CA, USA) (MS test) comprises of a set of prevalidated genotyping assays designed for the simultaneous detection of 128 *EGFR* gene mutations and 63 *KRAS*, *NRAS* and *BRAF* gene mutations using a PCR-based mass spectrometry method. This method uses a two-step reaction protocol in which DNA sequences of interest are first amplified by PCR, followed by a single base primer extension and termination reaction across variant nucleotide positions, before specific detection of the amplified allele-specific oligonucleotide reaction products by MALDI-TOF mass spectrometry. This method is now widely used for lung cancer mutation testing due to the need for rapid detection of an increasing number of therapeutically targetable genetic abnormalities across multiple lung cancer genes [20]. However, the MS-test is not yet approved by regulatory authorities for diagnostic use and limited data have been published on its clinical validity and utility [21].

With this background, this study sought to evaluate the clinical performance of the RT-PCR and MS tests in the setting of everyday testing of tumour specimens from lung cancer patients for *EGFR* gene mutations. To do so, tumour DNA extracts from a large and unselected group of lung cancer patients (n=532) previously tested by the RT-PCR test were retested by the MS test. Recently, we reported on the impact and uptake of *EGFR* gene mutation testing during the implementation of clinical practice guidelines for testing in this population of patients from northern New Zealand [1]. The clinical validity and utility of the tests were evaluated by agreement analysis and by correlating the test results with the treatment and survival outcomes of tested patients. These outcomes comprised of the patient overall survival and duration of benefit from EGFR-TKI treatment.

## RESULTS

### Study populations

Tumour DNA extracts from 532 NSCLC patients previously tested by the RT-PCR test were retested by the MS test (retested population). Valid results from both tests were available from 470 (88%) patients for an agreement analysis (agreement analysis population). Of 62 patients (12%) excluded from the agreement analysis, 2 (0.4%) had invalid MS test results, 9 (1.7%) had invalid RT-PCT test results and RT-PCR results were missing for 51 patients (10%). Survival data was available for analysis from 513 (96%) patients (survival population). The retested, agreement analysis and survival populations had similar demographic and clinical profiles (Table 1). Seventy-seven (14%) patients were treated with EGFR-TKIs (EGFR-TKI-treated population). The EGFR-TKI-treated population were younger, and had a higher proportion of females, Asians and Pacific people than the retested, agreement analysis or survival study populations (Table 1).

**Table 1:** Clinical characteristics and demographic factors of the retested, survival, agreement analysis and EGFR-TKI-treated study populations

Study population		Retested		Survival		Agreement analysis		EGFR-TKI-treated	
		N	%	N	%	N	%	N	%
Total		532		513		470		77	
	Median (range)	68.2(20.6-91.4)		67.4(20.6-91.4)		67.4(20.6-91.4)		63.3(40.9-86.8)	
Age	<60	117	22.0	117	22.8	105	22.3	27	35.1
	60-69	176	33.1	176	34.3	157	33.4	24	31.2
	70-79	157	29.5	157	30.6	143	30.4	18	23.4
	80+	63	11.8	63	12.3	56	11.9	7	9.1
	Unknown	19	3.6	0	0	9	1.9	1	1.3
Gender	Female	286	53.8	280	54.6	256	54.5	48	62.3
	Male	239	44.9	233	45.4	214	45.5	29	37.7
	Unknown	7	1.3	0	0	0	0	0	0
Ethnicity	NZ European	264	49.6	259	50.5	232	49.4	27	35.1
	NZ Maori	72	13.5	72	14.0	65	13.8	7	9.1
	Pacific	44	8.3	43	8.4	42	8.9	10	13.0
	Asian	63	11.8	63	12.3	60	12.8	21	27.3
	Other - mostly other European	74	13.9	74	14.4	65	13.8	11	14.3
	Unknown	15	2.8	2	0.4	6	1.3	1	1.3
Basis of diagnosis	Cytology or haematology	166	31.2	166	32.4	157	33.4	24	31.2
	Histology of primary	255	47.9	255	49.7	223	47.4	40	51.9
	Histology of metastasis	61	11.5	61	11.9	50	10.6	9	11.7
	Clinical investigation	5	0.9	5	1.0	5	1.1	1	1.3
	Death certificate	1	0.2	1	0.2	1	0.2	0	0.0
	Unknown	44	8.3	25	4.9	34	7.2	3	3.9
Extent	Localised to organ of origin	33	6.2	33	6.4	32	6.8	2	2.6
	Invasion of adjacent tissue or organ	24	4.5	24	4.7	24	5.1	4	5.2
	Regional lymph nodes	58	10.9	58	11.3	57	12.1	9	11.7
	Distant	262	49.2	262	51.1	226	48.1	47	61.0
	Unknown	155	29.1	136	26.5	131	27.9	15	19.5
Histology	Adenocarcinoma	432	81.2	432	84.2	388	82.6	66	85.7
	Others specified	22	4.1	22	4.3	19	4.0	2	2.6
	Not otherwise specified	29	5.5	29	5.7	24	5.1	5	6.5
	No pathological diagnosis	5	0.9	5	1.0	5	1.1	1	1.3
	Unknown	44	8.3	25	4.9	34	7.2	3	3.9
Time period	<2013	50	9.4	50	9.7	48	10.2	14	18.2
	2013	251	47.2	251	48.9	222	47.2	34	44.2
	2014	212	39.8	212	41.3	191	40.6	28	36.4
	Unknown	19	3.6	0	0	9	1.9	1	1.3
RT-PCR test result	*EGFR* gene mutation-positive	89	16.7	88	17.1	89	18.9	68	88.3
	*EGFR* gene mutation-negative	383	72.0	375	73.1	381	81.1	2	2.6
	Invalid or missing	60	11.3	50	9.7	0	0	7	9.1

### Agreement analysis

There was moderately high agreement between the RT-PCR test and the MS test for the detection of *EGFR* gene mutations. Among 470 patients with valid results available from both tests, 367 (78%) had no *EGFR* gene mutations detected in both tests and 71 (15%) had an *EGFR* gene mutation detected in both tests. The remaining 32 (7%) patients had an *EGFR* gene mutation detected by one test but not by the other test. From these data, the estimated levels of positive percentage agreement, negative percentage agreement and overall percentage agreement between the RT-PCR and MS tests for the detection of *EGFR* gene mutations were 79.8%, 96.9% or 93.2%, respectively (Table 2).

**Table 2:** Agreement analysis between the RT-PCR and MS test results for the detection of *EGFR* gene mutations in lung cancer patients

RT-PCR test	MS test		
	Mutation detected	No mutation detected	Total
Mutation Detected	71	18	89
No Mutation detected	14	367	381
Total	85	385	470
Positive percentage agreement		71/89 = 79.8% (95%CI; 70.3 to 86.8%)	
Negative percentage agreement		367/381 = 96.9% (95%CI; 93.9 to 97.8%)	
Overall percentage agreement		438/470 = 93.2% (95%CI; 90.5 to 95.1%)	

There was high agreement between the RT-PCR and MS test results for the identification of specific *EGFR* gene mutations among patients who had an *EGFR* gene mutation detected in both tests. Identical *EGFR* gene mutations were detected by both tests in 65 of 71 (92%) patient samples that had *EGFR* gene mutations detection by both tests (Table 3).

**Table 3:** Spectrum of *EGFR* gene mutations detected in 65 patients who were *EGFR* gene mutation-positive with identical mutations detected by both tests

*EGFR* gene mutation	Number of patients (%)
Exon 19 deletion	32 (49%)
L858R	28 (44%)
Exon 20 insertion	3 (5%)
G719X	2 (3%)
Total	65 (100%)

### Disagreement

As mentioned above, there was disagreement between the RT-PCR and MS test results for the detection of *EGFR* gene mutations in a total of 32 (7%) patients (Table 4). The RT-PCR test detected an *EGFR* gene mutation in 18 (4%) patients who had no *EGFR* gene mutations detected in the MS test. The RT-PCR test detected *EGFR* exon 20 insertions in 8 patients, exon 19 deletion mutations in 6 patients, exon 21 L858R mutations in 5 patients, and exon 20 point mutations in 2 patients, all of whom had no *EGFR* gene mutations detected by the MS test (Table 4). The MS test detected an *EGFR* gene mutation in 14 patients who had no *EGFR* gene mutations detected by the RT-PCR test. The MS test detected exon 21 L858R mutations in 4 patients, exon 20 insertion mutations in 3 patients, exon 19 deletion mutations in 3 patients, exon 20 point mutations in two patients, and an exon 19 insertion and an exon 21 point mutation in one patient each, all of whom had no *EGFR* gene mutations detected by the RT-PCR test (Table 4).

**Table 4:** Profiles of 32 discordant patient samples that had an *EGFR* gene mutation detected by one test but no *EGFR* gene mutations detected by the other test

		n
Sample type	Histology	23
	Cytology	9
RT-PCR Test DNA Quality Control check result	Valid	32
	Invalid	0
Specific EGFR and other oncogene mutations detected in discordant samples (RT-PCR result/MS result; NMD = no mutation detected)	Exon 20 Insertion/NMD	6
	Exon 19 Deletion/NMD	4
	Exon 21 L858R/NMD	3
	Exon 19 Deletion & Exon 20 S768I/NMD	1
	Exon 21 L858R & Exon 20 Insertion/NMD	1
	Exon 19 Deletion & Exon 20 T790M/KRAS G12C	1
	Exon 20 Insertion/BRAF V600E	1
	Exon 21 L858R/KRAS G12V	1
	NMD/EGFR L858R	4
	NMD/EGFR E746_A750del	2
	NMD/EGFR H773_V774insNPH	1
	NMD/EGFR D770_N771insSVD (5' Detection Only)	1
	NMD/EGFR D770_N771insG/D770_N771insGD (5' Detetion Only)	1
	NMD/EGFR T751I & KRAS G12V	1
	NMD/KRAS G12C & EGFR E746_A750del	1
	NMD/EGFR T751_I759>N	1
	NMD/EGFR L861Q	1
	NMD/EGFR L474_A750P (Rev Detection only),	1
Expected detectability of specific EGFR gene mutations identified in discordant samples	Detectable by both the RT-PCR and MS tests	27
	Detectable only by the MS test	5
	Detectable only by the RT-PCR test	0
Idylla Test DNA Quality Control check Result	Valid	26
	Invalid	3
	Not tested	3
Categorisation as true or false positive or negative test results according to Idylla retesting result (RT-PCR result/MS result)	True positive/false negative	9
	True negative/False positive	6
	False positive/True negative	7
	False negative/True positive	4
	Unknown	6

As in Table 4, all of the discordant samples passed the RT-PCR test DNA Quality Control check. Most (23 or 72%) were histological samples. Most of the specific *EGFR* gene mutations identified in the discordant samples were exon 19 deletions, exon 20 insertions or exon 21 L858R point mutations. Most of these specific *EGFR* gene mutations (27 or 84%) were expected to be detected by both the RT-PCR and MS tests.

The discordant patient samples were retested by a third assay (Biocartis Idylla *EGFR* Mutation Test) (Table 4). The Idylla test DNA Quality Control check was passed by all except three of the retested samples. The RT-PCR and MS test results were then re-categorised as true or false positive or negative tests according to the results of the third test (Table 4). Accordingly, nine patients had true-positive RT-PCR results and false-negative MS results; six patients had true-negative RT-PCR results and false-positive MS results; four patients had true-positive MS results and false-negative RT-PCR results, and; seven patients had true-negative MS results and false-positive RT-PCR results. Six discordant patients were not retested in the third assay because no specimen was available or failure of the DNA Quality Control check.

There was disagreement between the RT-PCR and MS tests for the identification of specific *EGFR* gene mutations in 6 of 71 (8%) patients who had *EGFR* gene mutations detected in both tests (Table 5). Four patients had double *EGFR* gene mutations detected by one test but only one mutation was detected by the other test. One patient had double mutations detected by both tests but one of those two mutations differed between the two tests. One patient had a single *EGFR* gene mutation detected by both tests but the specific mutation detected differed between the tests.

**Table 5:** Specific *EGFR* gene mutations detected in six *EGFR* gene mutation-positive patients in which different *EGFR* gene mutations were detected by the RT-PCR and MS tests

Patient	RT-PCR test	MS test
1	EGFR Exon 18 G719X and EGFR Exon 20 S768I	EGFR G719C
2	EGFR Exon 21 L858R	EGFR L858R and EGFR E709A
3	EGFR Exon 21 L858R	EGFR L858R and EGFR R108K
4	EGFR Exon 20 S768I and Exon 18 G719X	EGFR G719S and EGFR L861Q
5	EGFR Exon 18 G719X	EGFR G719S and EGFR L861Q
6	EGFR Exon 21 L858R	EGFR E709G

### *KRAS, NRAS* and *BRAF* gene mutation detection

The MS test identified a large number of patients with *KRAS, NRAS* and *BRAF* gene mutations that could not be expected to be detected by the RT-PCR test. Among 367 patients who had no *EGFR* gene mutations detected in both tests, a total of 127 patients (35%) had *KRAS, NRAS* and *BRAF* gene mutations identified by the MS test. The spectrum and frequency of specific *KRAS, NRAS* and *BRAF* mutations is shown in Table 6.

**Table 6:** Spectrum and frequency of specific *KRAS*, *NRAS* and *BRAF* gene mutations identified in *EGFR* gene mutation-negative patient samples by the MS test

RT-PCR test	MS assay	n
NMD	KRAS G12C	40
NMD	KRAS G12V	28
NMD	KRAS G12D	21
NMD	KRAS G12A	9
NMD	KRAS G13C	5
NMD	KRAS Q61H	4
NMD	BRAF V600E	3
NMD	KRAS G12R	2
NMD	KRAS Q61L	2
NMD	NRAS Q61R	2
NMD	BRAF G469R	1
NMD	KRAS A146T	1
NMD	KRAS G12C, KRAS G12V	1
NMD	KRAS G12S	1
NMD	KRAS G13D/N	1
NMD	KRAS Q61R	1
NMD	NRAS G12D/E, KRAS G12D	1
NMD	NRAS G13R	1
NMD	NRAS Q61H	1
NMD	NRAS Q61L	1
NMD	NRAS Q61Q/K	1

### Overall survival

**Figure 1:**
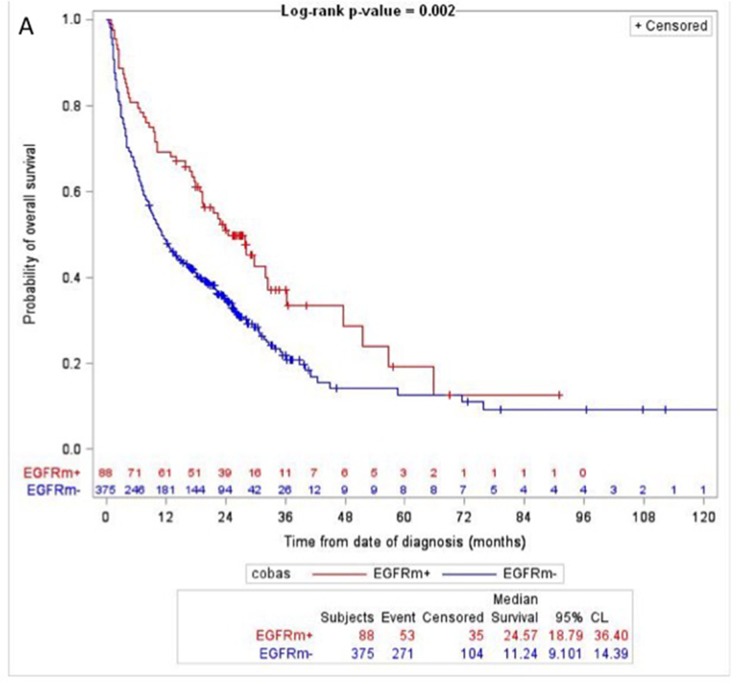

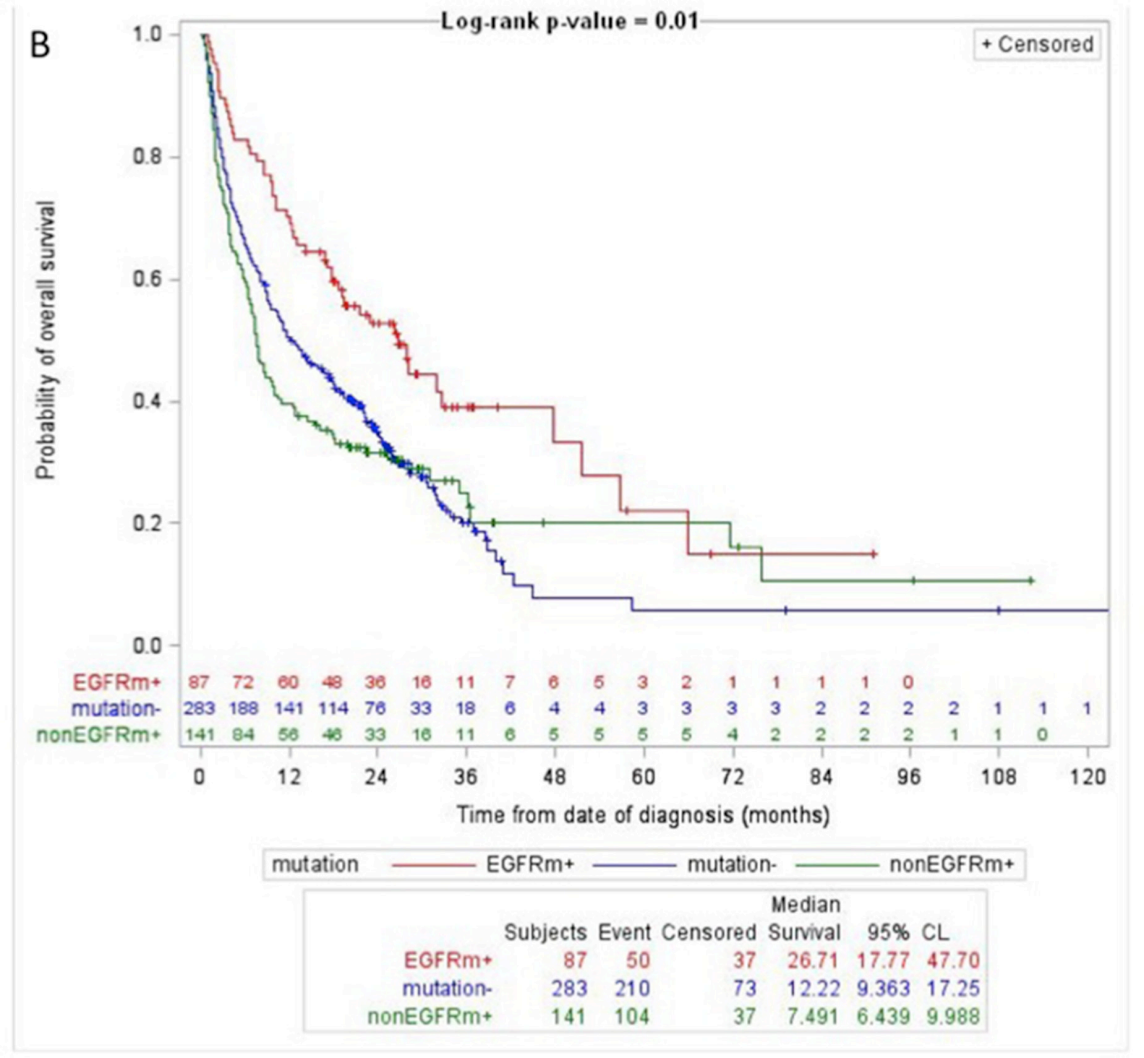

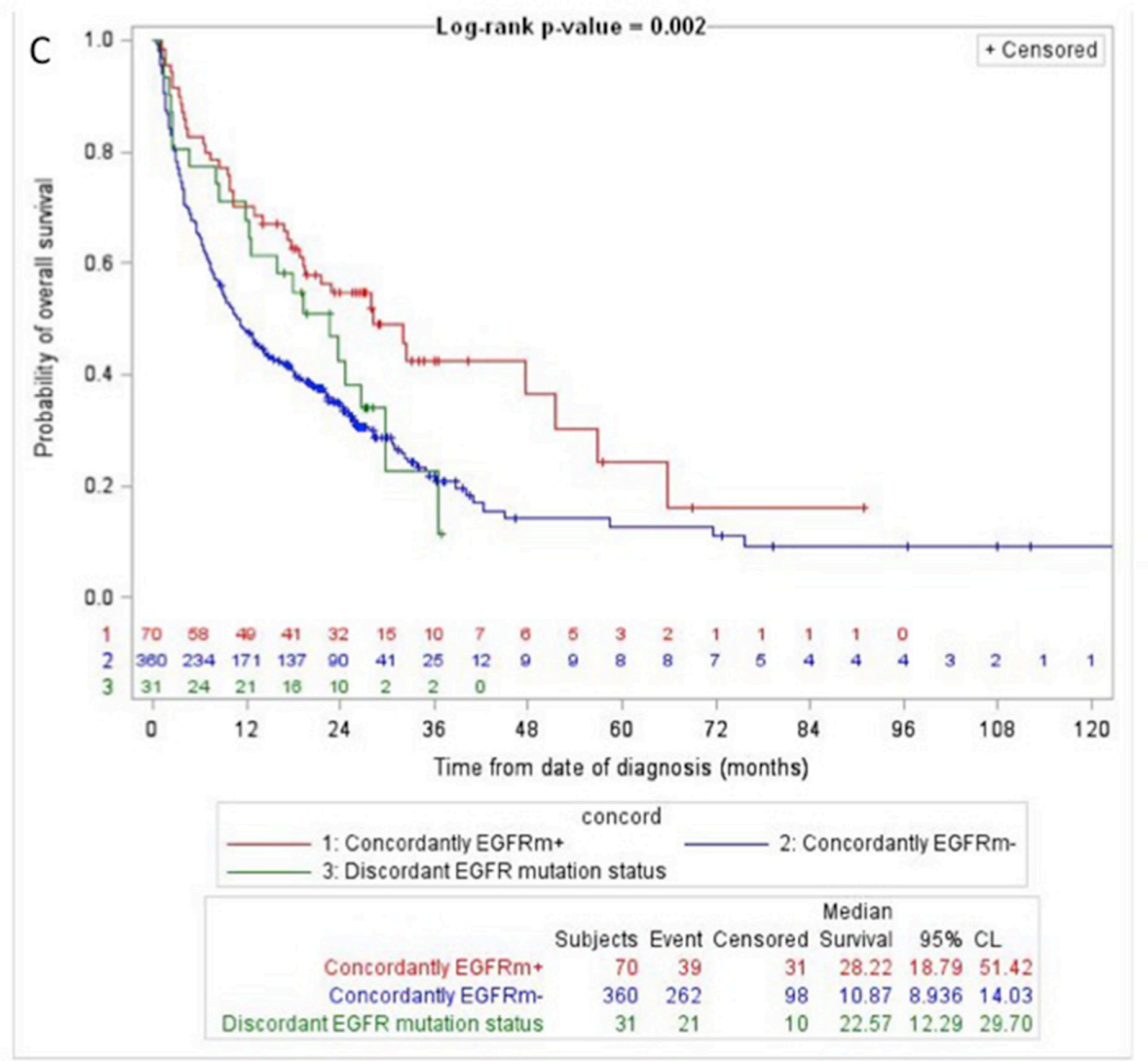
Overall survival by test results **(A)** RT-PCR test (Log-rank P-value = 0.002; *EGFR* gene mutation detected (EGFRm+, red); no *EGFR* gene mutations detected (EGFRm-, blue)). **(B)** MS test (Log-rank P-value = 0.01; *EGFR* gene mutation detected (EGFRm+, red); no mutations detected (mutation-, blue); *KRAS, NRAS* or *BRAF* gene mutation detected (nonEGFRm+, green)). **(C)** Agreement analysis (Log-rank P-value = 0.002; concordant detection of an *EGFR* gene mutation (Concordantly EGFRm+, red); concordant detection of no *EGFR* gene mutations (Concordantly EGFRm-, blue); discordant detection of an *EGFR* gene mutation by one test but not by the other test (Discordant EGFR mutation status, green)). Numbers at risk are shown above the x-axis.

Overall survival was correlated with the results of the RT-PCR test (Figure 1A), the MS test (Figure 1B) and agreement analysis (Figure 1C). The detection of an *EGFR* gene mutation was associated with significantly prolonged overall survival compared to when no *EGFR* gene mutations were detected, either by the RT-PCR test (adjusted HR=0.55 (95% CI 0.39 to 0.75))(Figure 1A), the MS test (adjusted HR=0.58 (95% CI 0.41 to 0.79))( Figure 1B) or by both tests (HR=0.47 (95% CI 0.32 to 0.66))(Figure 1C). The detection of a *KRAS, NRAS* or *BRAF* gene mutation was associated with a trend towards shorter overall survival compared to when no mutations were detected by the MS test (Figure 1B). The discordant detection of an *EGFR* gene mutation by one test but not the other test was associated with a trend for immediate overall survival compared to that of patients with concordantly positive or negative *EGFR* gene mutation tests (Figure 1C).

### Duration of EGFR-TKI treatment

**Figure 2:**
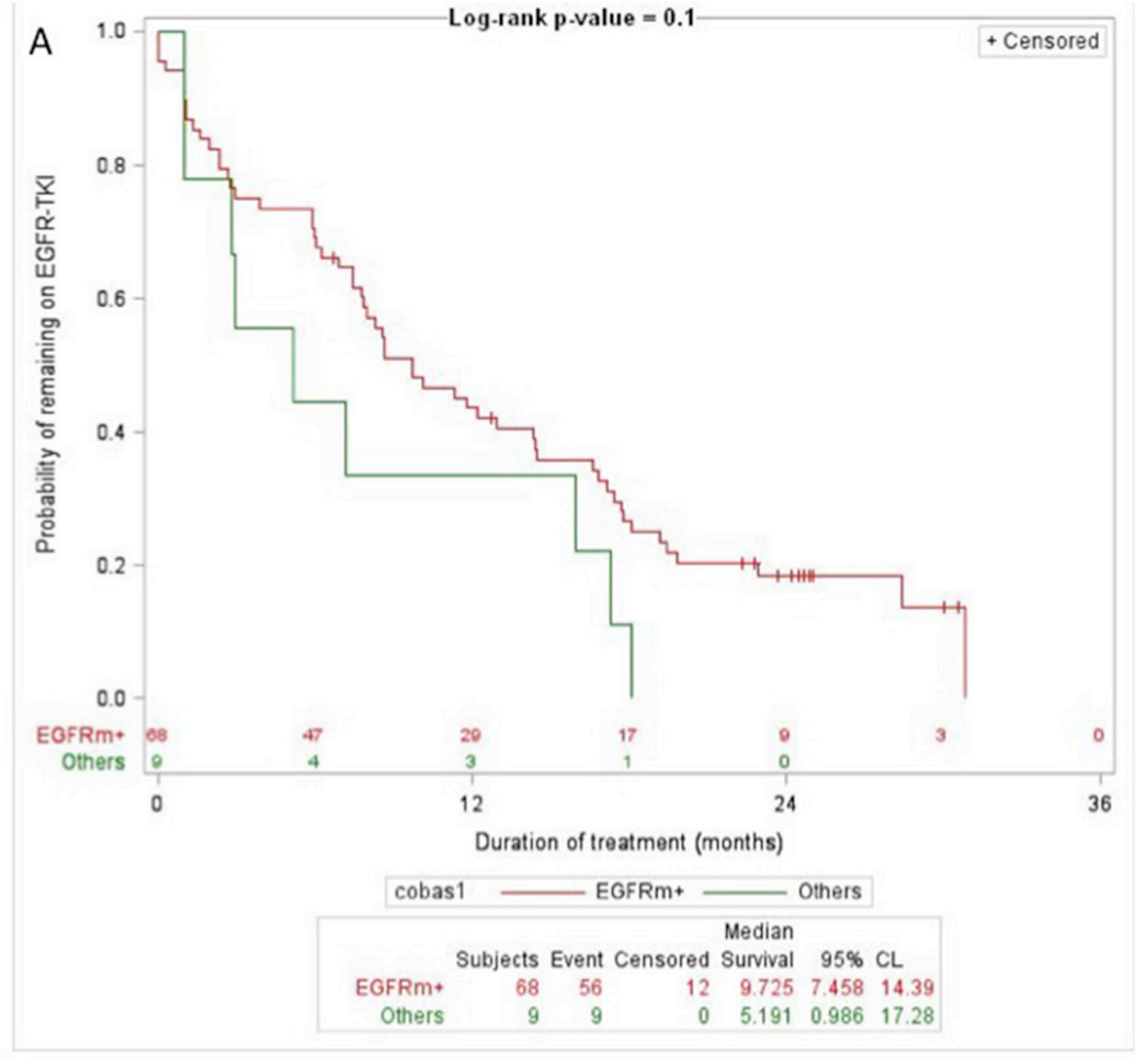

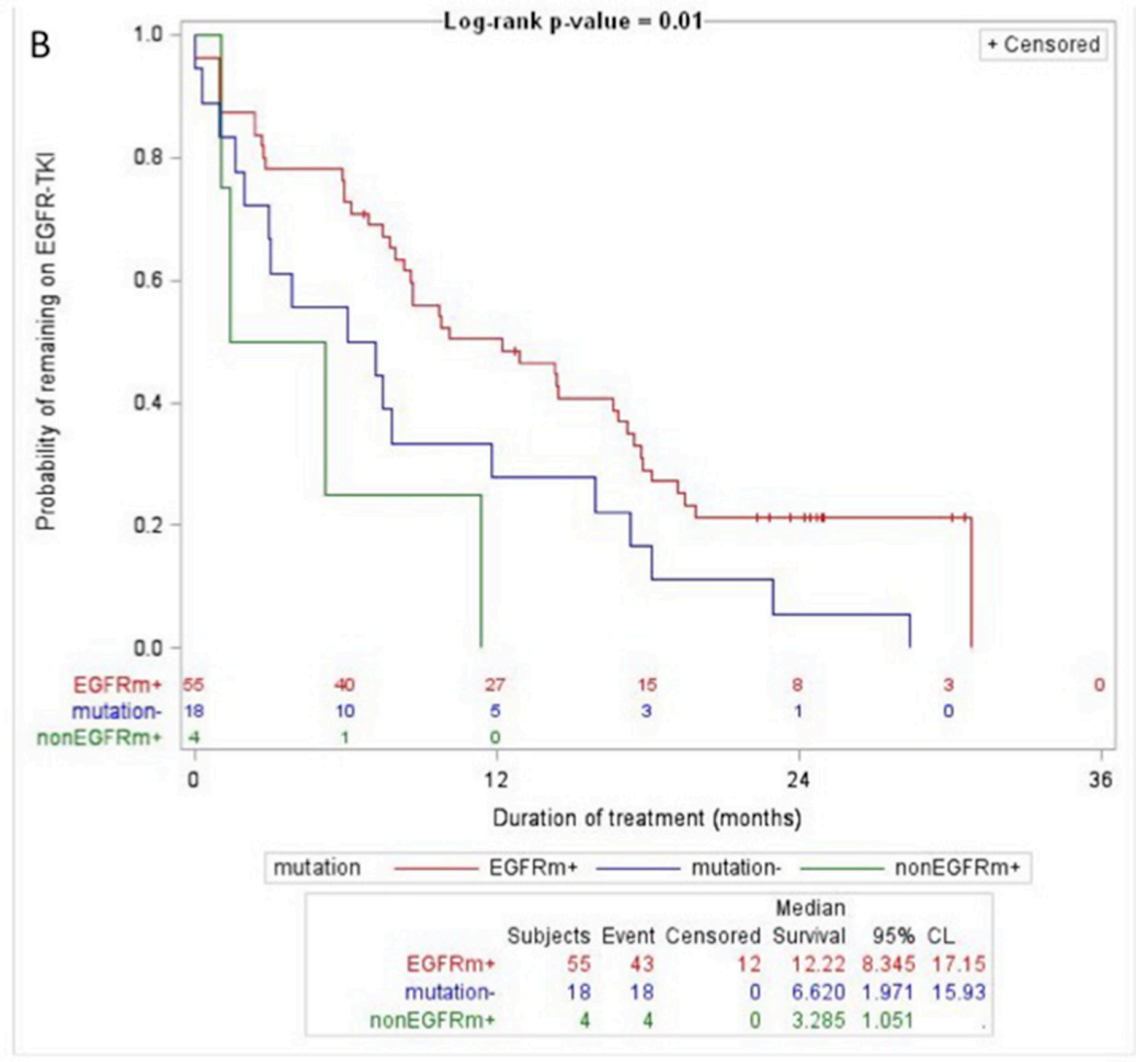

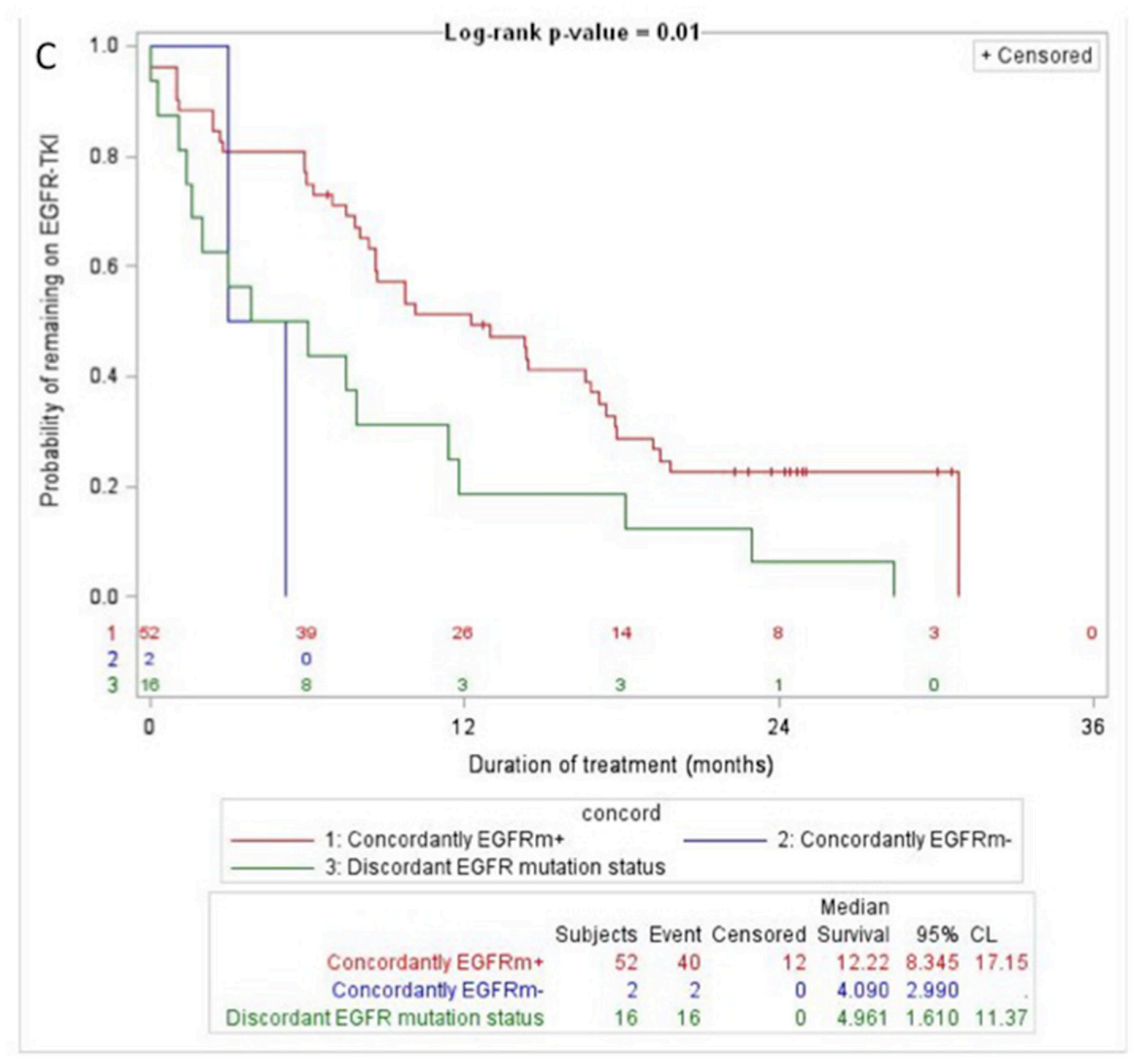
Duration of benefit from EGFR-TKI treatment by test results **(A)** RT-PCR test (Log-rank P-value = 0.1; *EGFR* gene mutation detected (EGFRm+, red); other results (Others, green)). **(B)**, MS test (Log-rank P-value = 0.01; *EGFR* gene mutation detected (EGFRm+, red); no mutations detected (mutation-, blue); *KRAS, NRAS* or *BRAF* gene mutation detected (nonEGFRm+, green)). **(C)** Agreement analysis (Log-rank P-value = 0.01; concordant detection of an *EGFR* gene mutation (Concordantly EGFRm+, red); concordant detection of no *EGFR* gene mutations (Concordantly EGFRm-, blue); discordant detection of an *EGFR* gene mutation by one test but not by the other test (Discordant EGFR mutation status, green)). Numbers at risk are shown above the x-axis.

The duration of benefit from EGFR-TKI treatment was correlated with the results of the RT-PCR test (Figure 2A), the MS test (Figure 2B) and agreement analysis (Figure 2C). The detection of an *EGFR* gene mutation was associated with trends toward longer duration of benefit from EGFR-TKI treatment compared to when no mutations were detected, either by the RT-PCR test (Figure 2A), the MS test (Figure 2B) or by both tests (Figure 2C). The detection of a *KRAS, NRAS* or *BRAF* gene mutation was associated with a trend toward shorter duration of benefit from EGFR-TKI treatment compared to when no mutations were detected by the MS test (Figure 2B). The discordant detection of an *EGFR* gene mutation by one test but not the other test was associated with a trend of intermediate duration of benefit from EGFR-TKI treatment compared to that of patients with concordantly positive or negative *EGFR* gene mutation tests (Figure 2C).

## DISCUSSION

The retesting study described here demonstrates moderately high agreement between a RT-PCR and a MS test for detecting *EGFR* gene mutations in lung cancer patients. The study was the first to directly compare these RT-PCR and MS tests despite both being commercially available and widely used in clinical practice for the detection of *EGFR* gene mutations in lung cancer patients. Both tests agreed in the detection of *EGFR* gene mutations in most patients with levels of positive, negative and overall agreement ranging from 79.8% to 96.9%. Identical *EGFR* gene mutations were detected by both tests in 92% of patients who were *EGFR* gene mutation-positive in both tests. In this way, this agreement study has provided evidence of the clinical validity of the RT-PCR and MS tests for detecting *EGFR* gene mutations in lung cancer patients.

However, this retesting study also demonstrates less than full concordance between the RT-PCR and MS tests for detecting *EGFR* gene mutations in lung cancer patients. The tests disagreed in the detection of *EGFR* gene mutations in some patients, with levels of positive, negative and overall disagreement ranging from 3.1% to 20.2%. The causes of this discordance were unclear but interesting to speculate upon. Most of the discordant results arose during the testing of small histological or cytological biopsy specimens, which are known to be less reliable for testing than surgical specimens [22]. The DNA quality control checks that were performed during the RT-PCR testing and Idylla retesting were passed by almost all of the discordant samples. The specific *EGFR* gene mutations identified in the discordant samples were most frequently exon 19 deletions, exon 20 insertions and exon 21 L858R point mutations, which are the most prevalent *EGFR* gene mutations associated with lung cancer [23] and most were expected to be detected by both the RT-PCR and MS tests. Oncogene mutation detection is known for being error-prone, due to, for example, contamination, PCR inhibitors and genetic polymorphisms. Contamination may occur during handling, DNA extraction or analysis [24, 25]. Reagents or reaction products used or produced during the testing may inadvertently inhibit PCR amplification [26]. Polymorphisms in genetic sequences targeted by primer and probe oligonucleotides used in a test protocol may also produce variation in PCR amplification [27, 28].

Retesting of the discordant samples by a third test showed that false-positive and false-negative test results were generated by both the RT-PCR and the MS test. Similar numbers of false-positive and false-negative results were produced by the RT-PCR and MS tests. Patients with discordant *EGFR* gene mutation test results had survival and treatment outcomes that were intermediate of patients with concordantly positive or negative *EGFR* gene mutation tests. While several factors may have influenced these intermediate outcomes, these findings suggest that some patients with discordant *EGFR* gene mutation test results survived and responded to EGFR-TKI treatment as would be expected of patients with true-positive *EGFR* gene mutation test results, whereas others did so as would be expected of patients with true-negative *EGFR* gene mutation test results. In these regards, false-positive and false-negative *EGFR* gene mutation test results may have important clinical consequences. If treatment decisions are based on false-positive results then such patients may receive less benefit from EGFR-TKI treatment but bear the same risks of adverse events compared to patients with true-positive *EGFR* gene mutation test results. Furthermore, if treatment decisions are based on false-negative test results then such patients may be denied substantial benefits of EGFR-TKI treatment, and bear increased risk of disease progression and death compared to patients with true-positive *EGFR* gene mutation test results. Other studies have also shown false-positive and false-negative results being generated by tests used for detecting EGFR gene mutations [29].

Despite less than full concordance, and false-positive and false-negative test results in some patients, treatment and survival outcomes of tested patients correlated with the results of the RT-PCR and MS tests in this study. The detection of an *EGFR* gene mutation by the RT-PCR or MS test was associated with significantly prolonged overall survival and trends toward more durable benefit from EGFR-TKI treatment, compared to when no *EGFR* gene mutations were detected. The demonstration of these associations with treatment and survival outcomes provides evidence of the clinical utility of the RT-PCR and MS tests for predicting the prognosis and clinical benefits of EGFR-TKI treatment in patients with lung cancer.

In conclusion, this study has provided evidence of the clinical validity and utility of a RT-PCR and a MS test for the detection of *EGFR* gene mutations that predict the prognosis and clinical benefits of EGFR-TKI treatment in lung cancer patients. However, caution and awareness are required regarding the possibility of false-positive and false-negative results being generated by these tests, which run the risk of important clinical consequences.

## MATERIALS AND METHODS

### Study design, context and population

The research described here was a clinical laboratory test-retesting study and clinical outcomes correlative study. Eligible patients were those referred to a New Zealand provider of *EGFR* gene mutation testing services between August 2012 and July 2014 (n=826), who had remnant tissue DNA extracts available for retesting after the completion of clinical diagnostic testing (n=532). The main study endpoint was the result of mutation testing and retesting. The main study outcomes were overall survival and the duration of treatment with an EGFR-TKI, either erlotinib or gefitinib. The Northern B Health and Disability Ethics Committee approved the study and required no individual patient consent. The study was registered with the Australian New Zealand Clinical Trials Registry (ACTRN12615000998549).

### Mutation testing

#### MS Test

The MS test was undertaken using the Sequenom/Agena MassARRAY System and Typer 4 software (Agena Bioscience, San Diego, CA, USA). The OncoFOCUS panel V1.0 was used following the manufacturer's instructions (OncoFOCUS^TM^ Panel Users Guide 1/8/2014). Testing was undertaken at the Auckland UniServices Sequenom facility, which was accredited (IANZ ISO 15189.2012) for clinical diagnostic *EGFR* molecular testing. Procedures undertaken to validate the test included establishing a low abundance control sample that demonstrated sensitivity for the detection of the *EGFR* L858R mutation to at least 10% mutation abundance. In addition, the facility participated in the Royal College of Pathologists Australasian Quality Assurance Programme involving blinded analysis of 69 clinical FFPE tumour samples, and an additional set of cell lines, both with known mutation profiles. These validation studies demonstrated 100% concordance with the expected results (Phillip Shepherd, Master of Medical Laboratory Science thesis submitted to Auckland University of Technology March 2017).

#### RT-PCR test

The RT-PCR test involved PCR amplification and detection of target DNA using complementary primer pairs and oligonucleotide probes. DNA was extracted using a Roche FFPE extraction kit and following manufacturer's instructions [15]; essentially this involved de-paraffinization and macro-dissection of tissue from slides previously marked by a histopathologist, thus ensuring the correct area was sampled. Following incubation in tissue lysis buffer, the lysate was bound to a filter column, washed and eluted. DNA was quantified using a Nanodrop® 1000 and diluted to 2 ng/μL prior to performing the RT-PCR test on the cobas® z 480 analyzer. The RT-PCR test consisted of three simultaneous PCRs encompassing 41 mutations within exons 18-21 of the *EGFR* gene. A mutant control and negative control were included in each run to confirm the validity of the run.

#### Third test

Patient samples with discordant results for the detection of *EGFR* gene mutations were retested by a third assay, the Biocartis Idylla EGFR Mutation Test [30]. The Idylla® System is an *in vitro* diagnostic (IVD) device, consisting of a console and instrument. The system uses disposable, single sample cartridges, in which paraffin embedded tissue is macro-dissected onto small filter paper circles which are inserted directly into a gene-specific cartridge. This is then placed in to the instrument, and all the processing steps take place within the hermetically-closed cartridge. The steps comprise liquefaction, cell lysis, DNA/RNA extraction, real-time amplification/detection, data analysis and reporting. Mutant and negative controls were incorporated into the cartridge. The *EGFR*-specific cartridge detects 52 mutations in exons 18-21 of the *EGFR* gene. The testing was conducted according to the manufacturer's instructions.

### Data sources

Mutation testing results were linked with other prospectively archived electronic data from healthcare administrative databases using individual patient-unique healthcare identifier numbers. Data extractions from the New Zealand Cancer Registry on tested patients provided information on the date of diagnosis and death, and codes for gender, ethnicity, place of domicile, morphology, basis of diagnosis, notifying laboratory, extent of disease, TNM, tumour grade and additional notes. Records of erlotinib and gefitinib dispensing were extracted from the Pharmaceutical Information database (PHARMS), which contains claims and payment information from pharmacists for subsidised dispensing in New Zealand, and was found in our pilot study to be a near-complete source of EGFR-TKI dispensing data. Where possible, electronic medical records were viewed to verify extracted data and provide additional information.

### Data analysis

Mutation testing results were tabulated by patient numbers and proportions were calculated according to whether or not a mutation was detected by each gene and by each specific mutation. The concordance between the mutation test results was analysed by agreement analysis [31]. A sample size of more than 400 patients was estimated to be sufficient for estimating proportions of agreement and population subgroups with 95% confidence intervals of less than 5%.

Cox proportional hazards regression modelling was used to assess hazards of overall mortality by the gene mutation status (*EGFR* gene mutation, *KRAS*, *NRAS* or *BRAF* gene mutation and no mutation) determined by the MS test and by agreement analysis results (concordant detection of an *EGFR* gene mutation by both RT-PCR and MS tests, discordant detection of an *EGFR* gene mutation by one test and not the other test, and no *EGFR* gene mutation). Overall survival was measured from the date of diagnosis to the date of death. Surviving patients were right-censored on 5 August 2016. Hazard ratios were adjusted by multivariate analysis for age, gender, ethnicity, histology, site and extent of disease, and diagnosis period.

Similar analyses were performed in a subgroup of patients who were treated with EGFR-TKIs and the duration of treatment was assessed against the gene mutation status determined by the MS test and by agreement analysis results. The duration of EGFR-TKI treatment was measured from the date that the drug was first dispensed to the date of the last dose (calculated as the date of last dispense plus the number of days of treatment dispensed on the last occasion) or death. Surviving patients who were continuing treatment with EGFR-TKIs were right-censored on 1 June 2016. No multivariate analysis was done because of the small sample sizes.

## Abbreviations

*EGFR*: Epidermal Growth Factor Receptor gene
EGFR-TKI: Epidermal Growth Factor Receptor-Tyrosine Kinase Inhibitor
FFPE: Formalin Fixed Paraffin Embedded
MALDI-TOF: Matrix Assisted Laser Desorption/Ionization-Time of Flight
MS test: Sequenom/Agena Biosciences MassArray OncoFocus mass spectrometry test
NSCLC: Non-Small Cell Lung Cancer
PCR: Polymerase Chain Reaction
RT-PCR test: cobas EGFR Mutation Test

## References

[1] McKeage M, Elwood M, Tin Tin S, Khwaounjoo P, Aye P, Li A, Sheath K, Shepherd P, Laking G, Kingston N, Lewis C, Love D (2017). EGFR mutation testing of non-squamous NSCLC: impact and uptake during implementation of testing guidelines in a population-based registry cohort from northern New Zealand. Target Oncol.

[2] Inoue A, Kobayashi K, Maemondo M, Sugawara S, Oizumi S, Isobe H, Gemma A, Harada M, Yoshizawa H, Kinoshita I, Fujita Y, Okinaga S, Hirano H (2013). Updated overall survival results from a randomized phase III trial comparing gefitinib with carboplatin-paclitaxel for chemo-naive non-small cell lung cancer with sensitive EGFR gene mutations (NEJ002). Ann Oncol.

[3] Maemondo M, Inoue A, Kobayashi K, Sugawara S, Oizumi S, Isobe H, Gemma A, Harada M, Yoshizawa H, Kinoshita I, Fujita Y, Okinaga S, Hirano H (2010). Gefitinib or chemotherapy for non-small-cell lung cancer with mutated EGFR. N Engl J Med.

[4] Mitsudomi T, Morita S, Yatabe Y, Negoro S, Okamoto I, Tsurutani J, Seto T, Satouchi M, Tada H, Hirashima T, Asami K, Katakami N, Takada M (2010). Gefitinib versus cisplatin plus docetaxel in patients with non-small-cell lung cancer harbouring mutations of the epidermal growth factor receptor (WJTOG3405): an open label, randomised phase 3 trial. Lancet Oncol.

[5] Rosell R, Carcereny E, Gervais R, Vergnenegre A, Massuti B, Felip E, Palmero R, Garcia-Gomez R, Pallares C, Sanchez JM, Porta R, Cobo M, Garrido P (2012). Erlotinib versus standard chemotherapy as first-line treatment for European patients with advanced EGFR mutation-positive non-small-cell lung cancer (EURTAC): a multicentre, open-label, randomised phase 3 trial. Lancet Oncol.

[6] Wu YL, Zhou C, Liam CK, Wu G, Liu X, Zhong Z, Lu S, Cheng Y, Han B, Chen L, Huang C, Qin S, Zhu Y (2015). First-line erlotinib versus gemcitabine/cisplatin in patients with advanced EGFR mutation-positive non-small-cell lung cancer: analyses from the phase III, randomized, open-label, ENSURE study. Ann Oncol.

[7] Zhou C, Wu YL, Chen G, Feng J, Liu XQ, Wang C, Zhang S, Wang J, Zhou S, Ren S, Lu S, Zhang L, Hu C (2015). Final overall survival results from a randomised, phase III study of erlotinib versus chemotherapy as first-line treatment of EGFR mutation-positive advanced non-small-cell lung cancer (OPTIMAL, CTONG-0802). Ann Oncol.

[8] Zhou CC, Wu YL, Chen GY, Feng JF, Liu XQ, Wang CL, Zhang SC, Wang J, Zhou SW, Ren SX, Lu S, Zhang L, Hu CP (2011). Erlotinib versus chemotherapy as first-line treatment for patients with advanced EGFR mutation-positive non-small-cell lung cancer (OPTIMAL, CTONG-0802): a multicentre, open-label, randomised, phase 3 study. Lancet Oncol.

[9] Mok TS, Wu YL, Thongprasert S, Yang CH, Chu DT, Saijo N, Sunpaweravong P, Han B, Margono B, Ichinose Y, Nishiwaki Y, Ohe Y, Yang JJ (2009). Gefitinib or carboplatin-paclitaxel in pulmonary adenocarcinoma. N Engl J Med.

[10] Han JY, Park K, Kim SW, Lee DH, Kim HY, Kim HT, Ahn MJ, Yun T, Ahn JS, Suh C, Lee JS, Yoon SJ, Han JH (2012). First-SIGNAL: first-line single-agent iressa versus gemcitabine and cisplatin trial in never-smokers with adenocarcinoma of the lung. J Clin Oncol.

[11] Lindeman NI, Cagle PT, Beasley MB, Chitale DA, Dacic S, Giaccone G, Jenkins RB, Kwiatkowski DJ, Saldivar JS, Squire J, Thunnissen E, Ladanyi M (2013). Molecular testing guideline for selection of lung cancer patients for EGFR and ALK tyrosine kinase inhibitors guideline from the College of American Pathologists, International Association for the Study of Lung Cancer, and Association for Molecular Pathology. J Thorac Oncol.

[12] Westwood M, Joore M, Whiting P, van Asselt T, Ramaekers B, Armstrong N, Misso K, Severens J, Kleijnen J (2014). Epidermal growth factor receptor tyrosine kinase (EGFR-TK) mutation testing in adults with locally advanced or metastatic non-small cell lung cancer: a systematic review and cost-effectiveness analysis. Health Technol Assess.

[13] Yatabe Y, Kerr KM, Utomo A, Rajadurai P, Tran VK, Du X, Chou TY, Enriquez MLD, Lee GK, Iqbal J, Shuangshoti S, Chung JH, Hagiwara K (2015). EGFR mutation testing practices within the Asia pacific region: results of a multicenter diagnostic survey. J Thorac Oncol.

[14] O'Donnell P, Ferguson J, Shyu J, Current R, Rehage T, Tsai J, Christensen M, Tran HB, Chien SS, Shieh F, Wei W, Lawrence HJ, Wu L (2013). Analytic performance studies and clinical reproducibility of a real-time PCR assay for the detection of epidermal growth factor receptor gene mutations in formalin-fixed paraffin-embedded tissue specimens of non-small cell lung cancer. BMC Cancer.

[15] Roche Molecular Systems Inc cobas® EGFR Mutation Test.

[16] Benlloch S, Botero ML, Beltran-Alamillo J, Mayo C, Gimenez-Capitan A, de Aguirre I, Queralt C, Ramirez JL, Cajal SRY, Klughammer B, Schlegel M, Bordogna W, Chen D (2014). Clinical validation of a PCR assay for the detection of EGFR mutations in non-small-cell lung cancer: retrospective testing of specimens from the EURTAC trial. PLoS One.

[17] Kimura H, Ohira T, Uchida O, Matsubayashi J, Shimizu S, Nagao T, Ikeda N, Nishio K (2014). Analytical performance of the cobas EGFR mutation assay for Japanese non-small-cell lung cancer. Lung Cancer.

[18] Lopez-Rios F, Angulo B, Gomez B, Mair D, Martinez R, Conde E, Shieh F, Tsai J, Vaks J, Current R, Lawrence HJ, de Castro DG (2013). Comparison of molecular testing methods for the detection of EGFR mutations in formalin-fixed paraffin-embedded tissue specimens of non-small cell lung cancer. J Clin Pathol.

[19] Wong AT, To RM, Wong CL, Chan WK, Ma ES (2013). Evaluation of 2 real-time PCR assays for *in vitro* diagnostic use in the rapid and multiplex detection of EGFR gene mutations in NSCLC. Diagn Mol Pathol.

[20] Kris MG, Johnson BE, Berry LD, Kawiatkowski DJ, Iafrate J, Wistuba II, Varella-Garcia M, Franklin WA, Aronson SL, Su PF, Shyr Y, Cambidge R, Sequist LV (2014). Using multiplexed assays of oncogenic drivers in lung cancers to select targeted drugs. JAMA.

[21] Okamoto I, Sakai K, Morita S, Yoshioka H, Kaneda H, Takeda K, Hirashima T, Kogure Y, Kimura T, Takahashi T, Atagi S, Seto T, Sawa T (2014). Multiplex genomic profiling of non–small cell lung cancers from the LETS phase III trial of first-line S-1/carboplatin versus paclitaxel/carboplatin: results of a West Japan Oncology Group study. Oncotarget.

[22] Fukui T, Ohe Y, Tsuta K, Furuta K, Sakamoto H, Takano T, Nokihara H, Yamamoto N, Sekine I, Kunitoh H, Asamura H, Tsuchida T, Kaneko M (2008). Prospective study of the accuracy of EGFR mutational analysis by high-resolution melting analysis in small samples obtained from patients with non-small cell lung cancer. Clin Cancer Res.

[23] Rosell R, Moran T, Queralt C, Porta R, Cardenal F, Camps C, Majem M, Lopez-Vivanco G, Isla D, Provencio M, Insa A, Massuti B, Gonzalez-Larriba JL (2009). Screening for epidermal growth factor receptor mutations in lung cancer. N Engl J Med.

[24] Zhang R, Han YX, Huang J, Ma L, Li YL, Li JM (2014). Results of first proficiency test for KRAS testing with formalin-fixed, paraffin-embedded cell lines in China. Clini Chem Lab Med.

[25] Asor E, Stav MY, Simon E, Fahoum I, Sabo E, Ben-Izhak O, Hershkovitz D (2017). Risk for molecular contamination of tissue samples evaluated for targeted anti-cancer therapy. PLoS One.

[26] Bessetti J (2007). A Introduction to PCR Inhibitors. https://worldwidepromegacom/resources/profiles-in-dna/2007/an-introduction-to-pcr-inhibitors/.

[27] Chen YL, Lu CC, Yang SC, Su WP, Lin YL, Chen WL, Huang WY, Su WC, Chow NH, Ho CL (2014). Verification of wild-type EGFR status in non-small cell lung carcinomas using a mutant-enriched PCR on selected cases. J Mol Diagn.

[28] Xu S, Duan Y, Lou L, Tang F, Shou J, Wang G (2016). Exploring the impact of EGFR T790M neighboring SNPs on ARMS-based T790M mutation assay. Oncol Lett.

[29] Min K, Kim W, Jang SJ, Choi YD, Chang S, Jung SH, Kim L, Roh MS, Lee CS, Shim JW, Kim MJ, Lee GK (2016). MassARRAY, pyrosequencing, and PNA clamping for EGFR mutation detection in lung cancer tissue and cytological samples: a multicenter study. J Cancer Res Clin Oncol.

[30] De Luca C, Gragnano G, Pisapia P, Vigliar E, Malapelle U, Bellevicine C, Troncone G (2017). EGFR mutation detection on lung cancer cytological specimens by the novel fully automated PCR-based Idylla EGFR Mutation Assay. J Clin Pathol.

[31] (2006). Guidance for Industry and FDA Staff - Statistical Guidance on reporting Results from Studies Evaluating Diagnostic Tests. U.S. Department of Health and Human Services Food and Drug Administration, Center for Drug Evaluation and Research (CDER), Center for Biologics Evaluation and Research (CBER)).

